# m^6^A Modification: A Key Epitranscriptional Regulatory Hub in Plant Reproductive Development

**DOI:** 10.3390/plants15213313

**Published:** 2026-10-26

**Authors:** Zhen Wen, Rui Sun, Xiaowen Da, Cheng Qin

**Affiliations:** 1 College of Life and Environmental Sciences, Hangzhou Normal University, Hangzhou 311121, China; 2023111010060@stu.hznu.edu.cn (Z.W.); 2025112010053@stu.hznu.edu.cn (R.S.); 2 College of Life Sciences, Zhejiang University, Hangzhou 310058, China

**Keywords:** m^6^A, plants, reproductive development

## Abstract

N^6^-methyladenosine (m^6^A) modification is a hallmark epitranscriptomic modification with dynamic and reversible properties in eukaryotes. The deposition, erasure, and recognition of m^6^A are coordinately regulated by methyltransferases (writers), demethylases (erasers), and binding proteins (readers), respectively. This review elaborates on the biological functions and molecular mechanisms of m^6^A in multiple reproductive and developmental processes of plants, including gametogenesis, embryogenesis, flowering, and seed setting. Numerous studies have verified that functional defects in core regulatory factors of m^6^A lead to severe developmental abnormalities during critical reproductive developmental stages in plants. On this basis, this paper further discusses the application prospects of crop improvement strategies centered on the m^6^A regulatory network. By dissecting the indispensable roles of m^6^A modification in plant reproduction and development, this study not only constructs a theoretical framework for clarifying the central functions of epitranscriptional regulation in germ cell fate determination and developmental timing control but also provides strategic references for the genetic improvement of crops.

## 1. Introduction

Organisms rely on reproduction to ensure the transmission of genetic and epigenetic information to the next generation and to maintain species continuity. The orderly progression of reproductive development depends on a complex and precise regulatory network, in which epigenetic determinants, such as DNA methylation, histone modifications, and RNA modifications, play critical regulatory roles in the dynamics of gene expression. As the most abundant internal chemical modification on eukaryotic mRNA [1], m^6^A plays an important role in the spatiotemporal regulation of numerous genes during core reproductive developmental processes.

m^6^A modification has been shown to be widely distributed across transcripts of plants such as rice (*Oryza sativa*), maize (*Zea mays*) [2], and *Arabidopsis* (*Arabidopsis thaliana*) [3], as well as mammals [4,5]. The m^6^A sites are predominantly enriched around stop codons, partial untranslated regions, and long exons of mRNAs, with RRACH (R = A/G, H = A/C/U) identified as its core conserved sequence.

m^6^A modification is a dynamic and reversible chemical modification. This dynamic process involves methylation mediated by methyltransferases (writers), demethylation catalyzed by demethylases (erasers), and the recognition and binding of m^6^A sites by m^6^A-binding proteins (readers). The methyltransferase complex is multi-subunit, ensuring the specificity and efficiency of methylation. Active demethylation by m^6^A demethylases can reverse m^6^A methylation, thereby reversing and controlling methylation-dependent processes. Through the recruitment of various readers, distinct regulatory or functional mechanisms can be directed to m^6^A-containing mRNAs, thereby influencing target mRNA splicing, stability, and translation [6,7].

During reproductive development, m^6^A participates in the regulation of key events such as gametogenesis, fertilization, and embryogenesis [8,9]. Notably, plants and animals exhibit significant differences in reproductive strategies, developmental processes, and environmental responses. As sessile organisms, plants undergo unique reproductive processes, including pollen development and double fertilization, and rely on photosynthesis to provide energy for reproductive development. Although research on m^6^A modification in plant reproductive development started later than that in animals, the critical role of m^6^A modification in regulating plant reproductive processes has garnered increasing attention. Based on this, this review systematically summarizes current research progress on m^6^A modification in plant reproductive development, focusing on three aspects: the composition and functional characteristics of the m^6^A regulatory machinery in plants, its regulatory roles and molecular mechanisms in key reproductive events (embryogenesis, flowering transition, gametogenesis, and fruit ripening), and the application prospects of m^6^A-based regulation in crop improvement. By dissecting the indispensable functions of m^6^A modification in plant reproduction, this review provides a theoretical framework for elucidating the central role of epitranscriptional regulation in developmental timing modulation and germ cell fate determination, as well as offering strategic references for crop genetic improvement.

## 2. Core Regulatory Mechanisms of m^6^A in Plants

**Figure 1:**
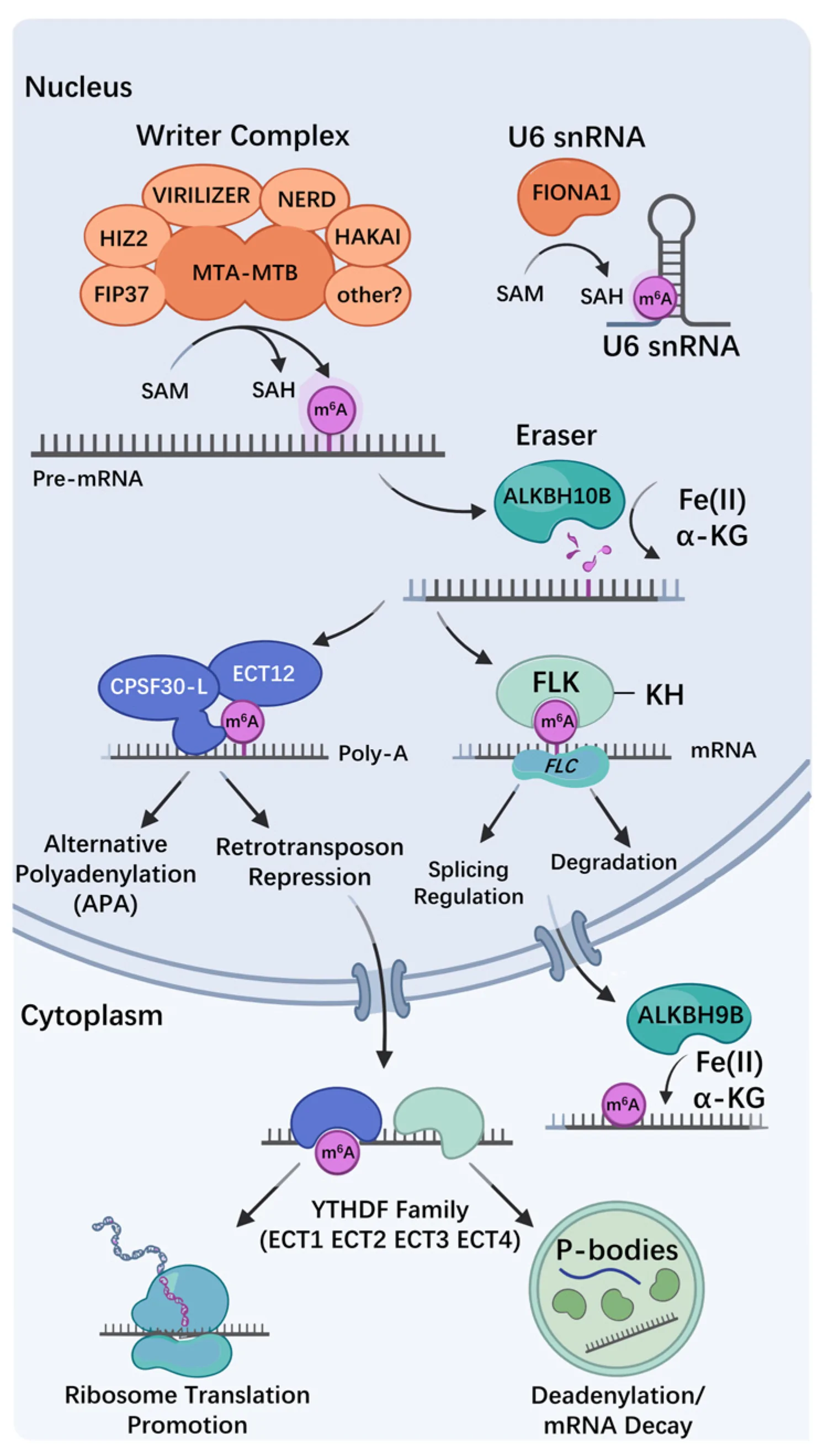
Regulatory components of m^6^A methylation in plant mRNA and their functions. In the nucleus, the m^6^A writer complex (MTA-MTB, FIP37, VIRILIZER, HAKAI, HIZ2, and NERD) deposits m^6^A on pre-mRNA, while FIONA1 independently modifies U6 snRNA. The Fe(II)/α-KG-dependent demethylases ALKBH10B (nuclear) and ALKBH9B (cytoplasmic) remove these marks. Nuclear readers CPSF30-L and ECT12 recognize m^6^A to regulate alternative polyadenylation (APA) and repress retrotransposons, whereas the KH-domain protein FLK mediates splicing regulation and mRNA degradation. In the cytoplasm, the YTHDF family (ECT1–ECT4) recognizes m^6^A-modified mRNAs to promote ribosome translation or facilitate deadenylation and decay within P-bodies. Note: light-blue shading marks the nucleus and the paler area the cytoplasm, separated by the nuclear envelope with nuclear pores. Solid arrows indicate the direction of metabolite flow or of the regulatory effect. Orange, teal and blue/pale-green ovals denote writer subunits, ALKBH erasers and reader proteins, respectively; ladder and hairpin symbols represent RNA, and pink circles mark m6A sites. The bilobed teal structure is the ribosome (emerging blue strand, nascent polypeptide) and the pale-green circle a P-body. “Other?” denotes putative, uncharacterized subunits. Abbreviations: m6A, N6-methyladenosine; SAM, S-adenosylmethionine; SAH, S-adenosylhomocysteine; APA, alternative polyadenylation; P-body, processing body; α-KG, α-ketoglutarate.

In plants, m^6^A methylation is a dynamic and reversible mRNA modification governed by writers, erasers, and readers, whose coordinated actions determine the deposition, removal, and functional decoding of m^6^A marks (Figure 1).

### 2.1. m^6^A Writer

In plants, m^6^A deposition on mRNAs is mediated by a conserved writer complex whose composition and division of labor are gradually being resolved (Figure 1). In *Arabidopsis thaliana*, the core subunits include MTA (methyltransferase A) and MTB, which are homologous to mammalian METTL3 (methyltransferase-like 3) and METTL14, respectively; MTA contains a canonical SAM (S-adenosylmethionine)-binding methyltransferase domain and forms the catalytic center, as does METTL3 in mammals [10,11,12,13,14,15,16]. FIP37 (FKBP12-interacting protein 37), homologous to mammalian WTAP (Wilms’ tumor 1-associating protein), maintains the protein levels of MTA and MTB, mirroring the role of WTAP in interacting with the METTL3–METTL14 core and maintaining full m^6^A activity in vivo [15,16,17,18,19,20,21]. VIRILIZER, homologous to mammalian VIRMA (Vir-like m^6^A methyltransferase associated), stabilizes the accumulation of MTA, MTB, FIP37, and HAKAI within the m^6^A methyltransferase complex; likewise, VIRMA interacts with the METTL3–METTL14 complex and is required for full m^6^A activity [17,18,19,20,22]. HAKAI is conserved and participates in the complete assembly of the complex by maintaining the stability of each constituent in both plants and mammals [15,16,21,23]. HIZ2 (HAKAI-interacting zinc finger protein 2) is another important plant factor affecting m^6^A abundance, and its interaction with MTA can occur independently of HAKAI [24]. This auxiliary layer is broadly reminiscent of mammalian RBM15 (RNA binding motif protein 15)/RBM15B (RNA binding motif protein 15B) and ZC3H13 (zinc finger CCCH domain-containing protein 13), which participate in m^6^A deposition and stabilize the WTAP–RBM15 interaction, respectively [19,25,26,27,28], although the plant auxiliary network appears to have diverged. NERD (NEEDED FOR RDR2-INDEPENDENT DNA METHYLATION), a recently reported plant-specific protein, is also an essential component of the m^6^A methyltransferase complex; it interacts with MTA and MTB and maintains their stability [29]. Disruption of genes encoding MTA, FIP37, VIR, HIZ2, or NERD leads to a pronounced global reduction in m^6^A modification (approximately 80–90% relative to wild-type levels). Together, these conserved and plant-specific modules facilitate stable and precise m^6^A deposition on target mRNAs.

In addition to the main complex that mediates the majority of m^6^A deposition in vivo, FIONA1 is responsible for low-level m^6^A regulation in plants. FIONA1, which is homologous to human METTL16, accounts for approximately 10–15% of m^6^A methylation in *Arabidopsis thaliana* in its monomeric form [30,31]. However, current studies remain inconsistent regarding the target range of FIONA1 [31,32]. Wang et al. [31] proposed that U6 snRNA is the primary substrate of FIONA1 based on in vitro enzyme activity assays and genetic analyses, whereas Xu et al. [32] identified more than 2000 mRNA targets by MeRIP-seq (methylated RNA immunoprecipitation sequencing), predominantly located in CDS regions. Subsequent studies verified that FIONA1 is indispensable for U6 snRNA methylation, and its deletion alters the global splicing pattern [33], implying that the thousands of targets identified by Xu et al. [32] may involve many indirect effects. Notably, although mammalian METTL16 also modifies U6 snRNA and recognizes the *hp1* hairpin within the 3′ UTR of *MAT2A* (SAM synthetase gene) to regulate *MAT2A* transcriptional processing and SAM homeostasis [30], FIONA1 fails to methylate the mRNA of the SAM synthetase gene [31]. This indicates that the regulatory mechanism of SAM homeostasis may have undergone functional divergence in plants.

### 2.2. m^6^A Eraser

In plants, m^6^A demethylation is mediated by AlkB family homologs, which are homologous to human ALKBH5 [34] (Table 1). Putative homologous genes in *Arabidopsis thaliana* include ALKBH9A, ALKBH9B, ALKBH9C, ALKBH10A, and ALKBH10B. Among these, ALKBH10B exhibits efficient oxidative reversal of m^6^A modifications on RNA both in vivo and in vitro, a feature associated with the regulation of flowering time in Arabidopsis [35]. Like human ALKBH5, ALKBH10B functions as an Fe(II)/α-KG-dependent dioxygenase, establishing m^6^A as a reversible mark [36]. ALKBH9B also exhibits m^6^A demethylase activity in vitro and can demethylate m^6^A sites on single-stranded RNA (Figure 1). Unlike human ALKBH5, which primarily localizes in the nucleus and acts on nuclear mRNAs, ALKBH9B is localized in the cytoplasm and is involved in mediating viral infection and transposition efficiency [35,37]. Similarly, ALKB family homologs with RNA m^6^A demethylation activity have been identified in other plant species, including OsALKBH5 [38] and OsALKBH9 [39] in rice (*Oryza sativa*), GhALKBH5 in cotton (*Gossypium hirsutum*) [40], FvALKBH10B in strawberry (*Fragaria vesca*) [41], SlALKBH2 in tomato (*Solanum lycopersicum*) [42], and AcALKBH10 in kiwifruit (*Actinidia chinensis*) [43]. In mammals, besides ALKBH5, FTO (fat mass and obesity-associated protein) was the first identified m^6^A demethylase; it also belongs to the AlkB family and preferentially targets m^6^A [34,44]. However, no clear FTO homolog has been identified in plants, suggesting that plant m^6^A demethylation relies primarily on ALKBH5 homologs.

### 2.3. m^6^A Reader

In eukaryotes, methyltransferases (writers) and demethylases (erasers) together determine the dynamic reversibility of m^6^A modification, while readers serve as downstream effectors that specifically recognize methylated sites and convert the chemical signal into functional outputs, thereby regulating various metabolic processes of m^6^A-modified RNAs, including splicing, transport, stability, degradation, storage, and translation [45,46].

In plants, m^6^A readers are also divided into YTH and non-YTH families, but unlike mammals, which possess diverse types of structurally distinct readers, plant readers are highly concentrated in the YTH family. Plant YTH domain-containing proteins are classified into two distinct subfamilies based on sequence similarity: YTHDF and YTHDC. In *Arabidopsis thaliana*, 13 YTH domain-containing proteins have been identified. Among them, 11 belong to the YTHDF subfamily; they are members of the evolutionarily conserved C-terminal (ECT) domain family and are named ECT1 to ECT11. These are mainly localized in the cytoplasm. The remaining two are nuclear-localized proteins: CPSF30 (CLEAVAGE AND POLYADENYLATION SPECIFICITY FACTOR 30) and ECT12 (*At4g11970*), which are homologous to YTHDC subfamily proteins [9,47,48]. This cytoplasmic/nuclear division resembles that of the mammalian YTH family, in which YTHDF1, YTHDF2, and YTHDF3 are cytoplasmically localized and primarily regulate RNA translation and stability [49,50], YTHDC1 is a nuclear protein involved in alternative splicing [51], and YTHDC2 is cytoplasmic and exhibits the weakest affinity for m^6^A among the YTH domain-containing proteins [52,53]. To date, among the plant YTH domain-containing family proteins, six have been demonstrated to possess m^6^A recognition activity, including ECT1, ECT2, ECT3, ECT4, a longer isoform of CPSF30 (CPSF30-L), and ECT12. ECT2 exhibits m^6^A recognition and binding activity on mRNAs both in vivo and in vitro (Figure 1). Subsequent genetic studies have indicated that ECT2, ECT3, and ECT4 are functionally redundant, and further experiments have confirmed their characteristics as m^6^A readers [54,55]. ECT1 is a newly identified m^6^A reader. In *Arabidopsis*, 96.66% of the mRNAs targeted by ECT1 are m^6^A-modified, and ECT1 functions to promote the degradation of m^6^A-modified mRNAs [56,57]. CPSF30-L and ECT12 function as nuclear m^6^A readers in plant cells. CPSF30 exists in two isoforms, with the longer isoform (CPSF30-L) containing a YTH domain and being the homolog of human YTHDC1. The YTH domain of CPSF30-L can bind to m^6^A in vitro and regulates the choice of polyadenylation sites in the 3′ UTRs of target mRNAs, thereby affecting mRNA stability and translational efficiency [58,59]. ECT12 directly binds to and regulates the stability of a set of m^6^A-modified stress-responsive gene mRNAs. Furthermore, ECT12 can directly interact with CPSF30-L to form a complex that co-binds m^6^A-modified retrotransposon RNAs (Figure 1), thereby suppressing retrotransposon transcription and expression [14,60].

In rice, 12 YTH domain-containing proteins have also been identified, among which OsYTH03, OsYTH05, and OsYTH10 have been confirmed to possess m^6^A recognition functions [61,62]. YTH domain-containing proteins have also been identified in other plant species, such as tomato [63], strawberry [64], and apple (*Malus domestica*) [65].

m^6^A readers in plants are not limited to the YTH family. FLK (FLOWERING LOCUS K) in *Arabidopsis thaliana* is a KH domain-containing protein localized in the nucleus and is homologous to the mammalian IGF2BP (insulin-like growth factor 2 mRNA-binding proteins) family (Figure 1). Mammalian IGF2BP family members recognize m^6^A through their KH (K homology) domains [66,67], and similarly, FLK recognizes m^6^A modifications in the 3′ UTR of mRNAs through its third KH domain, while simultaneously promoting target mRNA degradation and inhibiting its precursor splicing [68]. Beyond the YTH family, multiple non-YTH readers have been identified in mammals. The HNRNP (heterogeneous nuclear ribonucleoprotein) family contains several m^6^A-binding proteins with distinct modes of action: HNRNPA2B1 directly binds m^6^A via its RRM domain and participates in splicing regulation [69]; HNRNPC recognizes m^6^A indirectly through the so-called “m^6^A switch,” where m^6^A modification disrupts local RNA base pairing and exposes U-rich (uridine-rich) motifs [70,71,72]; and HNRNPR distributes across both the nucleus and cytoplasm and cooperates with IGF2BP1 to regulate mRNA stability and splicing [73,74]. Additional mammalian non-YTH readers include PRRC2A (proline-rich coiled-coil 2A) [75,76] and eIF3 (eukaryotic initiation factor 3) [77]. In contrast, non-YTH readers in plants remain less characterized, with FLK being one of the few clearly reported examples (Figure 1).

Collectively, the plant m^6^A regulatory machinery comprises a conserved writer core, an eraser system based mainly on ALKBH family homologs, and a reader network that is largely centered on YTH-family proteins but also includes non-YTH factors such as FLK. Compared with mammals, the writer and eraser layers show considerable conservation, whereas the reader layer and auxiliary components display notable plant-specific features, including NERD, HIZ2, and the functional divergence of FIONA1 from METTL16. These conserved and divergent components of the m^6^A regulatory machinery in humans and *Arabidopsis thaliana* are systematically compared in Table 1.

**Table 1:** Core components of the m^6^A regulatory machinery in humans and *Arabidopsis thaliana*.

Function	Representative Proteins in Humans	Localization	Representative Proteins in *Arabidopsis thaliana*	Localization
Writer (Core complex)	METTL3 [10,11]	Nucleus	MTA [15,21]	Nucleus
	METTL14 [18]	Nucleus	MTB [15]	Nucleus
	WTAP [18,19,20,25]	Nucleus	FIP37 [15,78]	Nucleus
	VIRMA [18,19,20]	Nucleus	VIRILIZER [15,22]	Nucleus
	ZC3H13 [19,20]	Nucleus	HIZ2 [24]	Nucleus
	HAKAI [20,23]	Nucleus	HAKAI [15]	Nucleus
Writer (Monomeric enzyme)	RBM15/RBM15B [26]	Nucleus	-	
	-		NERD [29]	Nucleus
	METTL16 [30]	Nucleus	FIONA1 [31]	Nucleus
Eraser (ALKB family)	ALKBH5 [34]	Nucleus	ALKBH9B [35]	Cytoplasm
			ALKBH10B [36]	Nucleus
	FTO [44]	Nucleus	-	
Reader (YTH family)	YTHDC1 [51]	Nucleus	CPSF30-L [58,59]	Nucleus
	YTHDC2 [53]	Cytoplasm, Nucleus	-	
	-		ECT12 [14,60] (YTHDC subfamily)	Nucleus
Reader (KH family)	IGF2BP1-3 [67]	Cytoplasm	FLK [68]	Nucleus
Reader (other)	HNRNPC [70]	Nucleus	-	
	HNRNPR [73]	Cytoplasm, Nucleus	-	
	PRRC2A [76]	Cytoplasm	-	

Note: “-” indicates that no clear orthologous protein has been identified.

### 2.4. m^6^A Detection Technologies in Plants

The ability to detect and quantify m^6^A modifications at the transcriptome level is fundamental to understanding their biological functions. In recent years, the continuous upgrading of high-throughput m^6^A sequencing technologies has enabled researchers to capture the dynamic alterations of m^6^A modifications during plant development with increasingly refined resolution. Based on differences in detection principles and application scenarios, plant m^6^A sequencing technologies can be roughly categorized into three types.

The first category consists of antibody-dependent transcriptome-wide detection technologies, represented by MeRIP-seq/m^6^A-seq (methylated RNA immunoprecipitation sequencing). This technology enriches modified RNA fragments using m^6^A-specific antibodies and is suitable for profiling the genome-wide distribution of m^6^A across the entire transcriptome. It stands as the most prevalently adopted method in current plant m^6^A research. Established for the first time in 2012, MeRIP-seq has made large-scale profiling of m^6^A modifications feasible [4]. In 2014, researchers successfully mapped the transcriptome-wide m^6^A modification landscapes of *Arabidopsis thaliana* and rice using m^6^A-seq, revealing that plant mRNAs contain an average of 2 to 3 m^6^A modification sites per transcript, which are predominantly enriched in the CDS regions, 3′ UTRs, and the vicinity of start codons [5,79,80]. Its derivative technology, miCLIP-seq (m^6^A individual-nucleotide resolution crosslinking and immunoprecipitation), can improve the resolution to nearly the single-base level via ultraviolet cross-linking [81]. Such technologies address the fundamental question of which gene transcripts carry m^6^A modifications. However, constrained by antibody specificity and sequencing depth, their resolution is generally limited to peak regions spanning 100 to 200 nucleotides, making it difficult to pinpoint individual modification sites with high precision [82].

The second category consists of antibody-independent quantitative technologies with single-base resolution, represented by m^6^A-SAC-seq (m^6^A-selective allyl chemical labeling and sequencing), which identifies and quantifies m6A sites at single-base resolution via chemical labeling or enzymatic reactions [83]. In 2024, researchers constructed transcriptome-wide m^6^A maps of different tissues in rice and *Arabidopsis* at single-base resolution using the m^6^A-SAC-seq technology. A total of 205,691 m^6^A sites were identified in rice, and 188, 282 m^6^A sites were identified in *Arabidopsis thaliana* [84]. Breakthroughs in such technologies have advanced relevant research from the qualitative level of whether modification exists or not to the precise quantitative level of which specific sites are modified and the modification abundance. Nevertheless, the application of these technologies in plants remains rather limited and is nowhere near the widespread adoption of MeRIP-seq. Related technologies also include DART-seq (deamination adjacent to RNA modification targets). This method tags m^6^A by fusing m^6^A-binding proteins with deaminases to induce C-to-U editing adjacent to modification sites [85].

The third category refers to direct single-molecule RNA sequencing technology, represented by DRS (Nanopore direct RNA sequencing) [86]. This technology enables direct sequencing of native RNA molecules without reverse transcription and amplification. It identifies m^6^A modifications by analyzing changes in electrical signals while simultaneously obtaining information on poly(A) tail lengths and transcript isoforms [87]. At present, Nanopore DRS has been applied in plants such as *Arabidopsis* [88,89] and wheat [90]. Nanopore direct RNA sequencing reveals N6-methyladenosine and polyadenylation landscapes on long non-coding RNAs in *Arabidopsis* [91]. Nevertheless, its application in plants is still in the initial stage due to existing challenges, including high costs, low throughput, complicated data analysis, and insufficient accuracy.

The iterative advancement of the three categories of technologies described above has progressively deepened researchers’ understanding of plant m^6^A modifications, from transcriptome-wide distribution maps to single-base-resolution modification sites.

## 3. Roles and Mechanisms of m^6^A in Plant Reproductive Development

**Figure 2:**
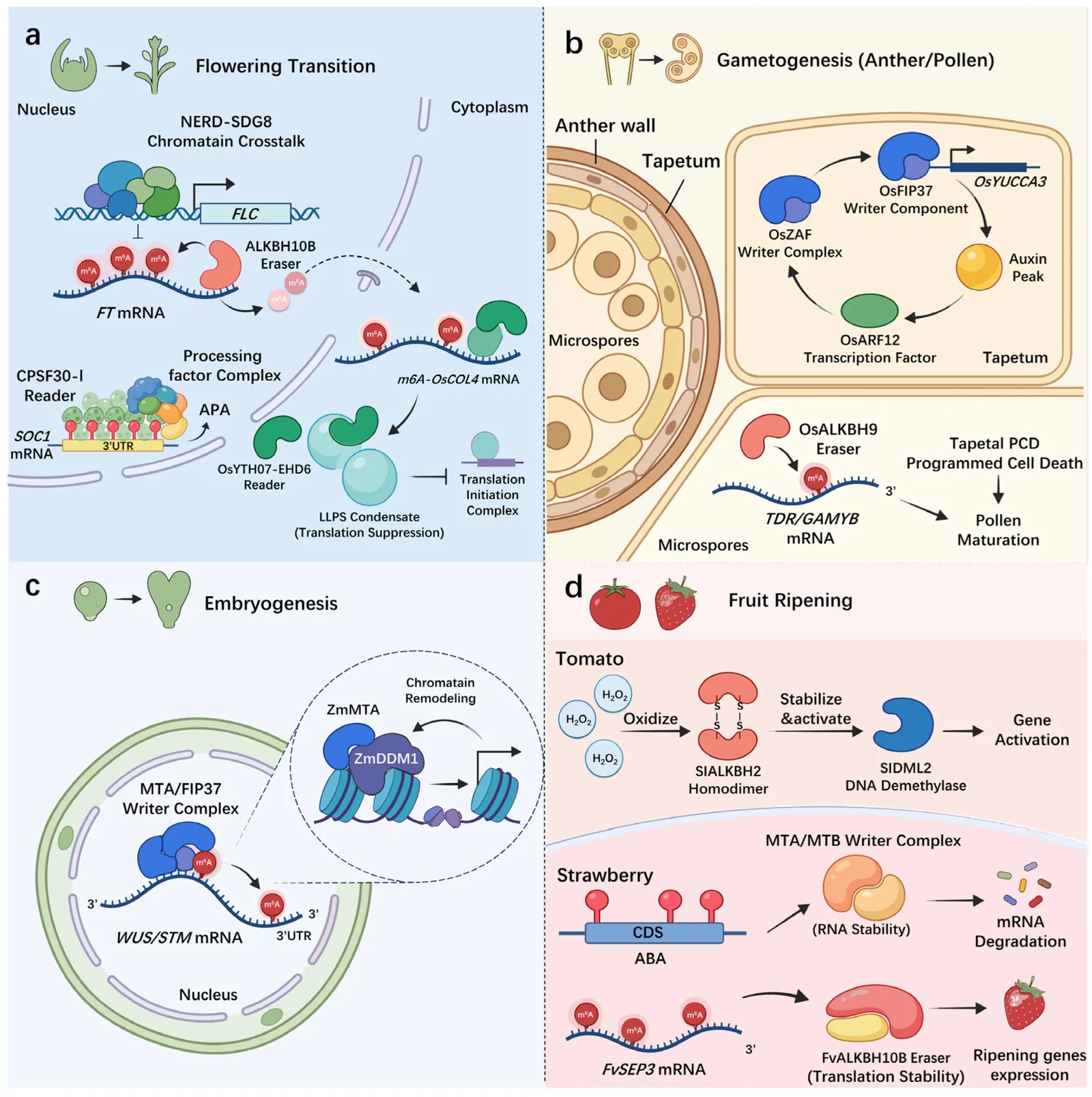
Roles and molecular mechanisms of m^6^A in plant reproductive development: (**a**) Flowering Transition: NERD-SDG8 mediates chromatin crosstalk at *FLC* to promote flowering, while ALKBH10B erases m^6^A from *FT* mRNA to enhance its stability and promote flowering. Additionally, the m^6^A reader CPSF30-L regulates APA of *SOC1* transcripts. with its loss extending the 3′ UTR and causing late flowering. OsYTH07-EHD6 sequesters m^6^A-OsCOL4 into LLPS condensates to suppress translation, promoting flowering. (**b**) Gametogenesis: The OsZAF-OsFIP37 complex modulates auxin peaks via the OsYUCCA3-OsARF12 loop to ensure normal meiosis, whereas OsALKBH9 erases m^6^A from *TDR/GAMYB* mRNA to promote tapetal PCD and pollen maturation. (**c**) Embryogenesis: The MTA/FIP37 writer complex deposits m^6^A on *WUS*/*STM* mRNAs to regulate shoot apical meristem maintenance, while ZmMTA interacts with ZmDDM1 to mediate chromatin remodeling during embryogenesis. (**d**) Fruit Ripening: In tomato, H_2_O_2_-induced SlALKBH2 homodimers stabilize SlDML2, leading to gene activation and promoting fruit ripening; in strawberry, MTA/MTB-mediated m^6^A modification regulates RNA stability of ABA pathway genes, while the FvALKBH10B eraser removes m^6^A from FvSEP3 mRNA to activate translation and promote ripening gene expression. Note: Solid arrows indicate promotion or progression to the next step; T-shaped bars indicate inhibition; curved arrows indicate recycling or enzymatic removal; and dashed curved arrows indicate transcript movement between the nucleus and the cytoplasm. Dashed straight lines linking a region to a circular inset mark a magnified view. Red circles labelled m6A mark m6A sites, navy strands represent transcripts, stacked discs represent nucleosomes, and scattered coloured fragments denote degraded mRNA. Colour distinguishes individual components rather than functional classes, each being identified by its adjacent label. Abbreviations: m6A, N6-methyladenosine; APA, alternative polyadenylation; LLPS, liquid–liquid phase separation; PCD, programmed cell death; CDS, coding sequence; UTR, untranslated region; H2O2, hydrogen peroxide.

m^6^A modification exhibits significant dynamic changes during plant reproductive development, with its modification abundance, transcriptome-wide distribution patterns, and tissue-specific sites all undergoing pronounced fluctuations across different developmental stages and reproductive phases. In *Arabidopsis thaliana*, for instance, MeRIP-seq data have shown that the overall m^6^A modification level in reproductive tissues is generally higher than that in vegetative tissues, and tissue-specific m^6^A sites are substantially enriched in regulatory pathways associated with embryogenesis and floral organ development [92]. Furthermore, single-base-resolution data from m^6^A-SAC-seq have revealed that tissue-specific m^6^A sites in rice reproductive tissues are predominantly concentrated in the 3′ UTRs, and their target genes are involved in core biological processes such as reproductive organ formation, embryogenesis, and photosynthesis [84]. Similarly, in animals such as mice (*Mus musculus*) and zebrafish (*Danio rerio*), deficiency of m^6^A regulatory factors leads to abnormalities including arrested follicle development, meiotic failure, and early embryonic lethality [93,94,95,96]. This evidence indicates that m^6^A modification serves as a core epitranscriptomic switch mediating temporal events in reproductive development (Figure 2).

### 3.1. Flowering Transition

For flowering plants, successful sexual reproduction and subsequent seed or fruit development depend on proper flowering time. The flowering transition is a key event in which plants shift from vegetative growth to reproductive growth. Core m^6^A regulatory components participate in the regulation of the flowering transition through multiple mechanisms.

#### 3.1.1. m^6^A Writer

In *Arabidopsis thaliana*, the m^6^A methyltransferase FIONA1 primarily modifies the coding sequence (CDS) region and mediates m^6^A modification of the key flowering-promoting gene *SOC1* (*SUPPRESSOR OF OVEREXPRESSION OF CONSTANS 1*) and its upstream regulators such as *SVP* (*SHORT VEGETATIVE PHASE*), thereby affecting their stability and preventing premature flowering [31,32]. NERD has been identified as a new component of the m^6^A writer complex. It specifically mediates m^6^A modification near the fourth intron of the nascent transcript of the flowering repressor *FLC* (*FLOWERING LOCUS C*). Furthermore, through its interaction with SDG8 (SET DOMAIN GROUP 8), an H3K36me3 methyltransferase, NERD negatively regulates H3K36me3 deposition and transcription at the *FLC* locus, thereby promoting flowering (Figure 2). This further reveals crosstalk between epitranscriptional and epigenetic regulation [29].

#### 3.1.2. m^6^A Eraser

In *Arabidopsis thaliana*, the demethylase ALKBH10B promotes flowering by removing m^6^A modifications from the mRNAs of *FT* (*FLOWERING LOCUS T*), *SPL3* (*SQUAMOSA PROMOTER BINDING PROTEIN-LIKE 3*), and *SPL9*, thereby enhancing their stability (Figure 2) [36]. Under heat stress, loss of ALKBH10B function represses the expression of heat-activated genes and reduces reproductive thermotolerance [92]. In cotton, the demethylase GhALKBH5 (a homolog of ALKBH5) is involved in regulating the expression of the photoperiodic flowering genes *GhADO3* (*ADVENTITIOUS ROOTLESS 3*) and *GhAGL24* (*AGAMOUS-LIKE 24*), revealing a role for m^6^A demethylation in the domestication of photoperiod sensitivity in cotton [40].

#### 3.1.3. m^6^A Reader

m^6^A readers are also involved in mediating the flowering transition. The rice reader OsYTH10 (YTH domain-containing protein 10) binds to the 3′ UTRs of *OsDTH7* (*DAYS TO HEADING 7*) and *OsGI* (*GIGANTEA*) mRNAs, enhancing their stability, thereby suppressing the expression of downstream *Ehd1* (*EARLY HEADING DATE 1*) and floral organ genes, and delaying flowering [62]. In contrast, the novel reader FLK (FLOWERING LOCUS K) in *Arabidopsis thaliana*, a KH domain-containing RNA-binding protein, promotes flowering by regulating the mRNA stability and alternative splicing of the flowering repressor *FLC* [68]. Furthermore, studies have revealed a novel mechanism by which m^6^A modification regulates flowering through phase separation. OsYTH07 interacts with the RNA-binding protein EHD6 (EARLY HEADING DATE 6) to form a complex that efficiently recognizes m^6^A-modified *OsCOL4* (*CONSTANS-like 4*) mRNA and sequesters it in ribonucleoprotein (RNP) granules via liquid–liquid phase separation (Figure 2), thereby suppressing its translation and promoting flowering [93].

In *Arabidopsis*, CPSF30-L primarily binds to the 3′ UTR of target mRNAs, recognizes the m^6^A-modified FUE (far-upstream element) and NUE (near-upstream element) signals, and forms dynamic droplets to facilitate the assembly of the polyadenylation complex, thereby regulating the choice of alternative polyadenylation (APA) sites (Figure 2). In the *cpsf30-l* mutant, global APA pattern alterations lead to 3′ UTR lengthening of numerous genes. Notably, the 3′ UTR of the flowering-promoting factor *SOC1* is significantly extended, accelerating its mRNA degradation and resulting in reduced transcript abundance, which ultimately leads to a late-flowering phenotype. However, how global APA changes mediated by CPSF30-L ensure the selective regulation of specific genes such as *SOC1* remains a question that merits further in-depth investigation. Although the m^6^A-binding function of CPSF30-L is required for the selection of proximal poly(A) sites [59], how the sequence and structural features surrounding m^6^A sites, as well as the composition of reader complexes, determine its differential regulation of different transcripts remains to be elucidated.

### 3.2. Gametogenesis

After floral transition, gametogenesis serves as the central hub of plant sexual reproduction, directly determining plant fertility, fertilization success rate, and the reproductive capacity of offspring. Male gametogenesis involves multiple key events, including meiosis, tapetal programmed cell death (PCD), and pollen wall formation [97], in which m^6^A modification exerts multilayered regulatory functions (Figure 2).

#### 3.2.1. m^6^A Writer

During rice meiosis, the methyltransferase OsFIP37 is highly expressed in anthers, and its loss of function leads to meiotic abnormalities and pollen abortion. OsFIP37 interacts with the RNA-binding protein OsFAP1 (FRIENDLY-ASSOCIATED PROTEIN 1) to specifically bind to the mRNA of *OsYUCCA3* (*YUCCA-LIKE GENE 3*), a key gene in auxin biosynthesis, and catalyzes m^6^A modification in its 3′ UTR, thereby stabilizing *OsYUCCA3* mRNA and promoting the formation of a local auxin peak, which is essential for normal meiotic progression (Figure 2) [98,99]. A recent study has further clarified the regulatory network: the C2H2-type zinc finger protein OsZAF (Zinc finger protein Activating FIP37 expression) specifically activates the transcription of *OsFIP37*, whereas auxin signaling upregulates *OsZAF* expression via the transcription factor OsARF12 (AUXIN RESPONSE FACTOR 12), thereby forming a positive feedback loop [OsZAF–OsFIP37–OsYUCCA3–OsARF12] that maintains the stability of the auxin peak in anthers during meiosis (Figure 2) [100]. Furthermore, the MTL (Methyltransferase-Like) domain-containing protein OsEDM2L (Enhancer of Dunce 2 Like) interacts with the transcription factors bHLH142 (basic Helix–Loop–Helix 142) and TDR (Tapetum Degeneration Retardation). By directly participating in the m^6^A modification of the *EAT1* (*Eternal Tapetum 1*) transcript, OsEDM2L influences its alternative splicing and alternative polyadenylation, ensuring the production of functional EAT1 protein, which in turn activates the downstream tapetal programmed cell death (PCD)-related genes OsAP25 (aspartic protease 25) and OsAP37. Mutation of *OsEDM2L* results in delayed tapetum degradation and microspore abortion [101]. Nevertheless, direct in vitro biochemical evidence is currently lacking to demonstrate that OsEDM2L itself possesses independent methyltransferase catalytic activity. Point mutation experiments confirmed that the MTL domain is required for its function but did not rule out the possibility that this domain functions by mediating protein–protein interactions. Therefore, whether OsEDM2L directly deposits m^6^A as a catalytic subunit or acts as an auxiliary factor to recruit other catalytic components remains to be distinguished by further biochemical experiments.

#### 3.2.2. m^6^A Eraser

During pollen wall formation and tapetum degradation, the m^6^A demethylase OsALKBH9 (AlkB Homolog 9) acts as a decelerator. It directly binds to the mRNAs of TDR and GAMYB (GA-MYB, a key transcription factor in gibberellin signaling) and removes their m^6^A modifications, thereby reducing their stability (Figure 2). In the *Osalkbh9-1* mutant, TDR and GAMYB mRNAs are hypermethylated and thus more stable, leading to overexpression of downstream genes involved in pollen wall synthesis and transport. This results in abnormally thickened pollen walls, delayed tapetal PCD, and ultimately male sterility (Figure 2) [39]. Another demethylase, OsALKBH5, is localized in the nucleoplasm and stabilizes the levels of mRNAs encoding multiple key meiotic genes, such as OsCOM1 (COM1/SAE2 homolog), OsZIP4 (Zinc transporter 4), and HEIP1 (HEI10 Interacting Protein 1), by removing their m^6^A modifications, thereby ensuring proper formation and repair of double-strand breaks (DSBs) during meiosis [38].

### 3.3. Embryogenesis

Following fertilization, embryogenesis initiates the next generation in the plant life cycle, and m^6^A methyltransferases are essential for this developmental stage.

In *Arabidopsis thaliana*, loss of function of MTA, a core component of the m^6^A methyltransferase complex, leads to embryonic developmental arrest at the globular stage, failure to progress to the heart stage, and abnormal endosperm development [15,21]. Similarly, knockout of MTB, FIP37, or VIR causes embryonic lethality, and loss of HIZ2 also results in severe developmental defects [24,78,102], indicating that the core m^6^A writer complex is indispensable for embryogenesis. In maize, ZmMTA is also critically important, as its mutation leads to severe defects in both embryo and endosperm development. Notably, ZmMTA is not only responsible for m^6^A deposition but also interacts with the chromatin remodeler ZmDDM1 (Domain-rearranged Methyltransferase 1), revealing a direct crosstalk between m^6^A modification and DNA methylation (Figure 2). This interplay may coordinately regulate gene expression during embryogenesis [103]. During embryogenesis, m^6^A modifications are mainly enriched near 3′ UTRs and stop codons [15,104]. The mRNAs of key embryonic development genes, such as *WUS* (*WUSCHEL*) and *STM* (*SHOOT MERISTEMLESS*), which function as shoot apical meristem maintenance factors, are regulated by m^6^A modification, affecting their stability and translational efficiency. For example, when FIP37 or MTA function is compromised, the mRNA stability of WUS and STM increases, leading to an expanded expression domain and overproliferation of the shoot apical meristem [78]. However, it should be noted that FIP37 and MTA are core components of the global m^6^A methyltransferase complex, and their loss of function affects the m^6^A modification status of thousands of transcripts, rather than being limited to *WUS* and *STM* (Figure 2). Genetic suppression experiments have shown that single mutations in *WUS* or *STM* only partially suppress the SAM overproliferation phenotype of *fip37* mutants [78], suggesting that the cooperative contributions of other m^6^A target genes cannot be ignored. Therefore, attributing SAM overproliferation primarily to dysregulation of *WUS* and *STM* may oversimplify the complexity of the m^6^A regulatory network. In the future, if site-specific m^6^A mutations could be introduced into the *WUS* and *STM* genomic loci without altering global m^6^A levels, this would more rigorously establish a direct causal chain.

### 3.4. Fruit Ripening

Fruit development and ripening constitute the final stage of the parental plant’s reproductive cycle, facilitating seed protection, maturation, and dispersal, thereby influencing species reproductive success and population spread [105]. m^6^A modifications play important regulatory roles in various fruit ripening processes, although the underlying mechanisms exhibit species specificity.

#### 3.4.1. m^6^A Writer

In strawberry, m^6^A modification undergoes dramatic changes at the onset of ripening, with a large number of new m^6^A peaks appearing in the coding sequence (CDS) region. This CDS hypermethylation is positively correlated with mRNA abundance. The methyltransferases MTA and MTB differentially regulate the stability or translational efficiency of key genes in the abscisic acid (ABA) signaling pathway (Figure 2), including NCED5 (9-cis-epoxycarotenoid dioxygenase 5), ABAR (ABA receptor), and AREB1 (ABA-responsive element binding protein 1), via m^6^A modification, thereby mediating ABA signaling to affect fruit ripening [106]. However, the reader proteins responsible for recognizing m^6^A sites in the CDS region of strawberry have not yet been identified, which is a notable gap in this field. m^6^A sites in the CDS region may achieve functional diversification through different reader protein complexes, but the specific mechanisms remain to be elucidated.

#### 3.4.2. m^6^A Eraser

In tomato, m^6^A modification levels globally decrease during ripening and are mainly enriched near stop codons and 3′ UTRs. The expression of the m^6^A demethylase SlALKBH2 (AlkB homolog 2) is regulated by DNA methylation. SlALKBH2 stabilizes the mRNA of the DNA demethylase gene *SlDML2* (*DEMETER-Like 2*), thereby promoting DNA demethylation in fruits and activating a series of ripening-related genes [42]. A recent study revealed that the SlALKBH2 protein can be oxidized by H_2_O_2_, forming homodimers via intermolecular disulfide bonds, which enhances its structural stability and thereby positively regulates fruit ripening (Figure 2). Conversely, NTRC (NADPH-dependent thioredoxin reductase C) catalyzes the reduction of SlALKBH2, delaying ripening. These findings uncover an intrinsic link between reactive oxygen species (ROS) signaling and epigenetic regulation [107]. Nevertheless, the concentration threshold and kinetic characteristics of H_2_O_2_ in this model remain unclear. The sensitivity of the oxidative modification of SlALKBH2 to H_2_O_2_ and whether endogenous H_2_O_2_ concentrations during normal ripening are sufficient to drive this modification still need to be confirmed by quantitative studies. In terms of flavor formation, the m^6^A reader proteins SlYTH1 (YTH domain-containing protein 1) and SlYTH2 play antagonistic roles. SlYTH1 binds to and stabilizes the mRNA of the gene encoding the flavor compound IBT (2-isobutylthiazole) synthase, thereby enhancing its translational efficiency and promoting aroma synthesis [108]. In contrast, SlYTH2 forms condensates via liquid–liquid phase separation, binds to the mRNAs of aroma synthesis genes, and suppresses their translation [109].

In kiwifruit, the m^6^A demethylase AcALKBH10 exhibits increased expression during ripening. It promotes ripening by removing m^6^A modifications from the mRNAs of ripening-related genes, such as ethylene receptors and sugar transporters. The function of AcALKBH10 has been validated by heterologous expression in tomato, indicating that this mechanism is conserved [43].

In strawberry, the m^6^A demethylase FvALKBH10B is directly activated by FvABF3 (ABA-responsive bZIP transcription factor 3), a transcription factor in the abscisic acid (ABA) signaling pathway. FvALKBH10B enhances the mRNA stability of the FvSEP3 (SEPALLATA 3, a MADS-box transcription factor) transcript by removing its m^6^A modifications, thereby initiating the expression of ripening-related genes (Figure 2) [41].

Overall, m^6^A modification exerts dynamic and stage-specific regulatory roles throughout the plant reproductive lifecycle, encompassing embryogenesis, flowering transition, gametogenesis, and fruit ripening. Among these, embryogenesis mainly relies on the m^6^A writer complex, whereas flowering transition, gametogenesis, and fruit ripening involve coordinated regulation by writers, erasers, and readers. Through modulating the stability, splicing, translation, and polyadenylation of key developmental transcripts, m^6^A ensures proper reproductive development and fertility. Table 2 systematically summarizes the diverse functions and fine-tuned regulatory mechanisms of m^6^A in these processes.

**Table 2:** Functions of m^6^A modification in major reproductive events of plants.

Reproductive Event	Species/Regulator	Core Target	m^6^A-Mediated Process on Target RNA	Main Phenotype/Function
Male gametogenesis	Rice: OsALKBH5 [38]	Key genes in meiosis	Stability	Ensures DSB formation and repair during meiosis
	Rice: OsALKBH9 [39]	*TDR*, *GAMYB*	Stability	Prevents abnormal pollen exine thickening
	Rice: OsFIP37 [98,99]	*OsYUCCA3*	Stability	Maintains local auxin peak, ensures normal meiosis
	Rice: OsEDM2L [101]	*EAT1*	Alternative splicing	Produces functional EAT1 protein, promotes tapetum PCD
Embryogenesis	*Arabidopsis*: MTA FIP37 [21,78]	*WUS*, *STM*	Stability	Restricts shoot meristem size
	*Arabidopsis*: NERD [29]	*FLC* nascent transcript	Co-transcriptional m^6^A, recruits H3K36me3	Represses *FLC* transcription, promotes flowering
	*Arabidopsis*: FIONA1 [32]	*SOC1*, *SVP*	Stability	Prevents early flowering
	*Arabidopsis*: ALKBH10B [36]	*FT*, *SPL3*, *SPL9*	Stability	Promotes flowering
	Cotton: GhALKBH5 [40]	*GhADO3*, *GhAGL24*	Stability	Promotes flowering
	*Arabidopsis*: CPSF30-L [59]	*SOC1*	Alternative polyadenylation	Regulates flowering time
	Rice: OsYTH10 [62]	*OsDTH7*, *OsGI*	Stability	Delays flowering
	Rice: OsYTH07 [110]	*OsCOL4*	Translation	Promotes flowering
	Strawberry: FvALKBH10B [41]	*FvSEP3*	Stability	Activates ripening-related gene expression
	Tomato: SlALKBH2 [42]	*SlDML2*	Stability	Promotes fruit ripening
	Kiwifruit: AcALKBH10 [43]	Ethylene receptors, sugar transporters	Stability	Promotes fruit ripening
	Strawberry: FvMTA-FvMTB [106]	*NCED5*, *ABAR*, *AREB1*	Stability Translation	Promotes fruit ripening
	Tomato: SlYTH1 [108]	Flavor-volatile synthesis genes	Stability Translation	Enhances aroma synthesis
	Tomato: SlYTH2 [109]	Aroma synthesis genes	Translation	Negatively regulates fruit aroma

## 4. Applications of m^6^A Modification in Crop Improvement

Based on the mechanistic insights discussed above, which highlight the essential fine-tuning functions of m^6^A modification in plant reproductive development, exploring its translational applications in crop improvement has emerged as a highly promising research direction.

Male sterile lines are a core breeding tool for exploiting heterosis in crops. Studies have shown that hybrid yields can be 15–50% higher than those of inbred lines [111]. Strategies for creating novel male sterile lines based on m^6^A modification have begun to show promise. In rice, *OsALKBH9* encodes an mRNA m^6^A demethylase. Knockout of *OsALKBH9* using CRISPR/Cas9 technology leads to complete and stable male sterility, and this sterile phenotype depends on its m^6^A demethylase activity [39]. In addition to OsALKBH9, other m^6^A regulatory factors that can induce male sterility have also been identified in rice, including the methyltransferase OsFIP37 [98], the MTL domain-containing protein OsEDM2L [101], and the demethylase OsALKBH5 [38]. These factors may serve as potential molecular targets for generating novel male sterile lines in rice. The flowering transition is a key developmental event in which plants shift from vegetative growth to reproductive growth. Through the synergistic actions of writers [29], erasers [36], and readers [68], m^6^A modification exerts multilayered core regulatory functions in the flowering regulatory network. The elucidation of these mechanisms provides molecular targets for the targeted improvement of crop adaptability and regional yield. For example, regulating the m^6^A reader OsYTH10 can modulate the flowering process in rice under long-day conditions [62].

m^6^A modification is involved in regulating fruit ripening and quality in tomato [108], kiwifruit [43], and strawberry [41]. These findings suggest that, in the future, precise editing of m^6^A modification levels on specific ripening-related genes could enable directional improvement of quality traits such as flavor and color while maintaining normal fruit development, thereby meeting diverse market demands.

Furthermore, a representative success in cross-species yield enhancement has been achieved: transgenic expression of the human m^6^A demethylase FTO in rice and potato led to approximately 50% increases in yield and biomass in field trials, without altering nutritional quality. This effect was consistently observed across different genetic backgrounds and multiple independent transgenic lines [112]. This strategy demonstrates the broad potential of modulating m^6^A modification to achieve effective crop yield improvement.

On the technical support front, the Cotton Genetic Improvement Team at Huazhong Agricultural University has developed a targeted m^6^A editor, fusing the CRISPR/dCas13 (Rx) system with the m6A methyltransferase GhMTA (TME) and the demethylase GhALKBH10 (TDE), respectively. This system enables the addition or removal of m^6^A modifications at specific sites on specific transcripts without altering the DNA sequence, providing a new tool for precise crop trait improvement [113]. Meanwhile, single-base resolution sequencing technologies such as m^6^A-SAC-seq enable the genome-wide identification of key m^6^A sites associated with fertility in various crops [84], thereby offering precise targets for cross-species crop improvement strategies.

## 5. Conclusions and Perspectives

### 5.1. Conclusions

In plants, m^6^A modification is dynamically regulated by a conserved writer complex, AlkB-family erasers, and a reader apparatus dominated by the YTH family [36,48,54,114]; continuous innovations in m^6^A detection technologies have driven increasingly in-depth functional studies [115]. Although the core enzymatic machinery is evolutionarily conserved across eukaryotes, plant reader proteins exhibit pronounced YTH family diversification, and these m^6^A readers can also participate in the post-transcriptional regulation of target RNAs through diverse regulatory modes [116,117]. This suggests that during the evolution of m^6^A readers, plants may have adapted to the environmental variability and developmental plasticity demands associated with their sessile lifestyle by expanding the YTH family. Functionally, m^6^A regulates embryogenesis, flowering transition, gametogenesis, and fruit ripening, and impaired function of m^6^A regulatory components often leads to severe reproductive defects [29,39,78,106]. These mechanistic insights also hold practical potential, as modulating m^6^A pathways is expected to improve crop yield and quality. In summary, m^6^A serves as a central epitranscriptional regulatory hub in plant reproduction and a highly promising target for future crop improvement.

### 5.2. Perspectives

Despite considerable progress in plant m^6^A research in recent years, significant knowledge gaps and technical bottlenecks remain in the following areas, warranting focused investigation in the coming years.

#### 5.2.1. Single-Cell Resolution of m^6^A Dynamics

Key events in plant reproductive development, such as microsporogenesis, embryo sac formation, and zygote activation, involve highly heterogeneous cell types, many of which are extremely scarce in number. Conventional bulk RNA sequencing masks the transcriptomic specificity of these rare cell populations due to signal averaging [118,119]. In recent years, single-cell and single-nucleus omics technologies have advanced rapidly in plant research; scRNA-seq (single-cell RNA sequencing) has been widely adopted, and chromatin accessibility assays (scATAC-seq) are also being progressively extended [120]. These approaches have been successfully applied in crops, including rice, wheat, peanut, and soybean, providing unprecedented resolution for dissecting cell-type-specific mechanisms underlying plant development, cell identity, and stress responses [118,121]. However, single-cell m^6^A sequencing has not yet been established in plants. Although technologies such as scm^6^A-seq have enabled simultaneous profiling of the m^6^A methylome and transcriptome at the single-cell level in animal oocytes and early embryos [122], the adaptation of single-cell epitranscriptomic methods in plants remains challenging due to technical obstacles, including the difficulty of cell wall digestion and the low efficiency of protoplast isolation [123].

Future efforts should focus on developing single-cell m^6^A sequencing methods compatible with plant protoplasts and rare reproductive cells in order to uncover cell-type-specific m^6^A modification dynamics and their regulatory networks during germline development [124]. For instance, loss-of-function of core m^6^A writer components such as MTA, MTB, FIP37, or VIR leads to embryonic lethality in *Arabidopsis* [15,21,24], which precludes the in-depth dissection of the specific regulatory mechanisms and effects of m^6^A during embryogenesis. If m^6^A sequencing could be performed at the single-cell level on sperm cells, zygotes, and individual lineages of early embryos, it would greatly facilitate the elucidation of stage- and lineage-specific m^6^A dynamics and their target regulatory events, thereby refining our understanding of the functional roles of m^6^A at critical developmental junctures such as zygotic activation and embryonic patterning.

#### 5.2.2. The Directionality and Causal Hierarchy of m^6^A–Epigenome Crosstalk Remain Largely Unclear

In recent years, substantial evidence has accumulated in plants linking m^6^A to histone modifications [125,126]. For example, in *Arabidopsis thaliana*, the m^6^A writers FIP37 and VIR mediate methylation of retrotransposon RNAs. Upon recognition by the nuclear readers CPSF30-L and ECT12, they recruit the histone methyltransferases SUVH4/5/6 and ATXR5/6 to deposit H3K9me2 and H3K27me1 at transposon loci, thereby establishing a closed heterochromatin state [14]. Furthermore, the newly identified writer component NERD negatively regulates H3K36me3 levels at the flowering repressor *FLC* locus by interacting with the H3K36me3 methyltransferase SDG8, thereby promoting flowering [29]. In maize, the core writer ZmMTA directly interacts with the chromatin remodeler ZmDDM1; m^6^A-modified genes exhibit significantly higher DNA methylation levels than non-m^6^A-modified genes, and loss of ZmMTA function results in a marked decrease in CHH methylation in the 5′ regions of m^6^A-modified genes [103]. In animals, H3K36me3 has been shown to guide co-transcriptional deposition of m^6^A, while m^6^A can in turn influence histone modification status through the recruitment of enzymes such as SETDB1 [127].

Nevertheless, in these regulatory interactions, it remains unclear whether m^6^A modification acts as an upstream signal driving the recruitment of histone-modifying enzymes or merely reflects an already established chromatin state. Current correlative evidence is insufficient to distinguish causality from coincidence. For instance, NERD-mediated m^6^A modification occurs concomitantly with reduced H3K36me3 deposition [29], but the temporal order and causal direction between these two events have not been validated through dynamic tracking experiments. Similarly, in the ZmMTA–ZmDDM1 interaction in maize, loss of ZmMTA leads to a decrease in CHH methylation [103]. However, whether this is a direct consequence of reduced m^6^A or an indirect effect remains to be resolved. Recent studies have revealed bidirectional crosstalk between m^6^A and histone modifications—namely, that m^6^A can influence histone modifications [128], and histone modifications can in turn affect m^6^A deposition [129], which further compounds the complexity of causal interpretation. Systematic perturbation experiments are urgently needed to disentangle cause and effect. These include (i) utilizing inducible m^6^A depletion systems to remove m^6^A modifications at specific developmental time points and subsequently tracking the dynamic changes in histone modifications [130]; (ii) combining time-series m^6^A sequencing with ChIP-seq to capture the temporal order of the two modifications over the course of the same developmental process [129]; and (iii) employing site-specific m^6^A editing tools to add or remove m^6^A at defined sites on specific transcripts and observing the resulting local changes in histone modifications [113]. These approaches, when integrated, will greatly facilitate the transition from correlative observations to causal mechanistic understanding of the m^6^A–epigenome interplay.

#### 5.2.3. m^6^A Editing Tools and Precision Breeding

RNA m^6^A editing tools based on the CRISPR-Cas system have advanced rapidly in recent years. By fusing m^6^A methyltransferase or demethylase domains with catalytically dead Cas9/Cas13 variants, a series of programmable RNA m^6^A editors have been developed and successfully applied in mammalian cells, stem cells [131], and plant systems [132]. In plants, researchers have established programmable m^6^A editing tools [113]. In addition, light-inducible m^6^A editing systems, engineered by combining CRISPR-dCas13 proteins and sgRNAs with light-inducible heterodimerization modules, have achieved efficient and reversible m^6^A level modulation with minimal off-target effects [133]; these tools have been applied to manipulate major regulatory genes involved in flowering time, stress adaptation, and yield.

Nevertheless, to realize their potential in precision breeding, several technical bottlenecks must be addressed, including further improvement of targeting efficiency and specificity, systematic evaluation of off-target effects, precise control of the editing window, and stable transgenerational inheritance of m^6^A editing-conferred traits [132,134]. Future efforts should aim to establish more efficient platforms for directed evolution of m^6^A effectors [135], develop m^6^A editing delivery systems applicable to diverse plant species and tissues, and explore novel strategies integrating m^6^A editing with synthetic biology. Furthermore, the integration of m^6^A editing technology with other breeding approaches, such as gene editing and marker-assisted selection, will open new avenues for precision design breeding in crops.

## Data Availability

No new data were created or analyzed in this study. Data sharing is not applicable to this article.
